# A randomized comparison of an adhesive gelatin sponge and a plain collagen sponge for hemostatic control during canine liver surgery

**DOI:** 10.1111/vsu.14160

**Published:** 2024-10-08

**Authors:** Thomas S. Anderson, Rachel D. Hattersley, Jackie L. Demetriou

**Affiliations:** ^1^ Dick White Referrals Station Farm Cambridgeshire UK; ^2^ School of Veterinary Medicine and Science University of Nottingham Loughborough UK

## Abstract

**Objective:**

To compare the effectiveness of a modified surface gelatin sponge to a plain collagen sponge for hemostasis of parenchymal hepatic bleeding.

**Study design:**

Prospective, randomized trial of two hemostatic agents.

**Animals:**

A total of 45 dogs undergoing elective liver surgery were randomly allocated into two groups: 22 in the adhesive gelatin (AG) group and 23 in the plain collagen (PC) group. A total of 20 patients per group underwent liver biopsy to create a uniformly sized bleeding surface, with the remaining patients (AG = 2, PC = 3) undergoing liver lobectomy.

**Methods:**

Evaluation of hemostatic effectiveness and tissue adhesion of each sponge type was performed by the operating surgeon using structured scoring systems. Hemostatic parameters were primarily evaluated at the liver biopsy site to maintain homogeneity of bleeding surface size.

**Results:**

For the liver biopsy group (*n* = 40), 5 min after hemostatic sponge application, 10/20 dogs were bleeding in the PC group, compared to 2/20 in AG group (*p =* .0138). The PC bleeding was significantly higher than AG across the 3 to 6 min evaluation period (*p* < .001). When surgeons tested the adhesion of the sponge across the whole cohort (*n* = 45), AG scored 2 (of 3) against 1 for PC (*p* < .001). In group PC, 5/23 sponges dislodged during abdominal lavage and preparations for closure and had to be replaced due to recurrence of bleeding, compared with no AG sponges dislodging (*p =* .042). There were no further complications related to the use of either sponge.

**Conclusion:**

In the dogs with hepatic parenchymal incision, use of an adhesive gelatin sponge improved intraoperative attachment and haemostatic effectiveness, compared to a collagen sponge.

**Clinical significance:**

Based on our clinical experience in these cases, adhesive gelatin sponges could be considered an effective option when selecting a hemostatic agent for liver surgery in dogs.

## 1 INTRODUCTION

Mechanical hemostatic agents such as gelatins, collagens, and cellulose are commonly utilized in canine hepatic surgery.^1^ Due to the friable nature of liver parenchyma, traditional methods of hemostasis such as ligatures and vascular clips are less likely to be efficacious.^1^ Mechanical hemostatic sponges work by providing a matrix for platelet aggregation and activation, clot formation and stabilization, which then provides a barrier or tamponade to prevent ongoing hemorrhage.^1^, ^2^, ^3^, ^4^

In the authors' practice, a bovine‐derived collagen sponge (Lyostypt, B. Braun, Germany) is the hemostatic sponge of choice for canine hepatic surgery. In our experience, this collagen matrix provides sufficient hemostasis while held in place. However, we have noted inconsistent adhesion of the collagen sponge, resulting in sponge dislodgement and subsequent bleeding from the cut surface of the liver. The human market has moved in the direction of the adoption of adhesive hemostatic sponges to reduce the risk of dislodgement associated postoperative bleeding.^2^, ^5^, ^6^, ^7^, ^8^, ^9^ Most adhesive sponges contain human blood‐derived products, for example Tachosil (Corza Medical, USA) is a human market leader containing human thrombin and fibrinogen.^6^, ^8^, ^9^ These agents are costly, can be challenging to store, and may lead to adverse reactions both in humans and veterinary patients.^9^ Therefore, there is interest in the human market into adhesive hemostatic sponges which do not contain human‐derived blood products. The adhesive and tissue sealant market is expected to register the largest growth in the future, due to increased adoption of advanced surgical procedures that require efficient tissue sealing solutions. The human market size is already in excess of 2 billion US dollars.^9^, ^10^

**FIGURE 1:**
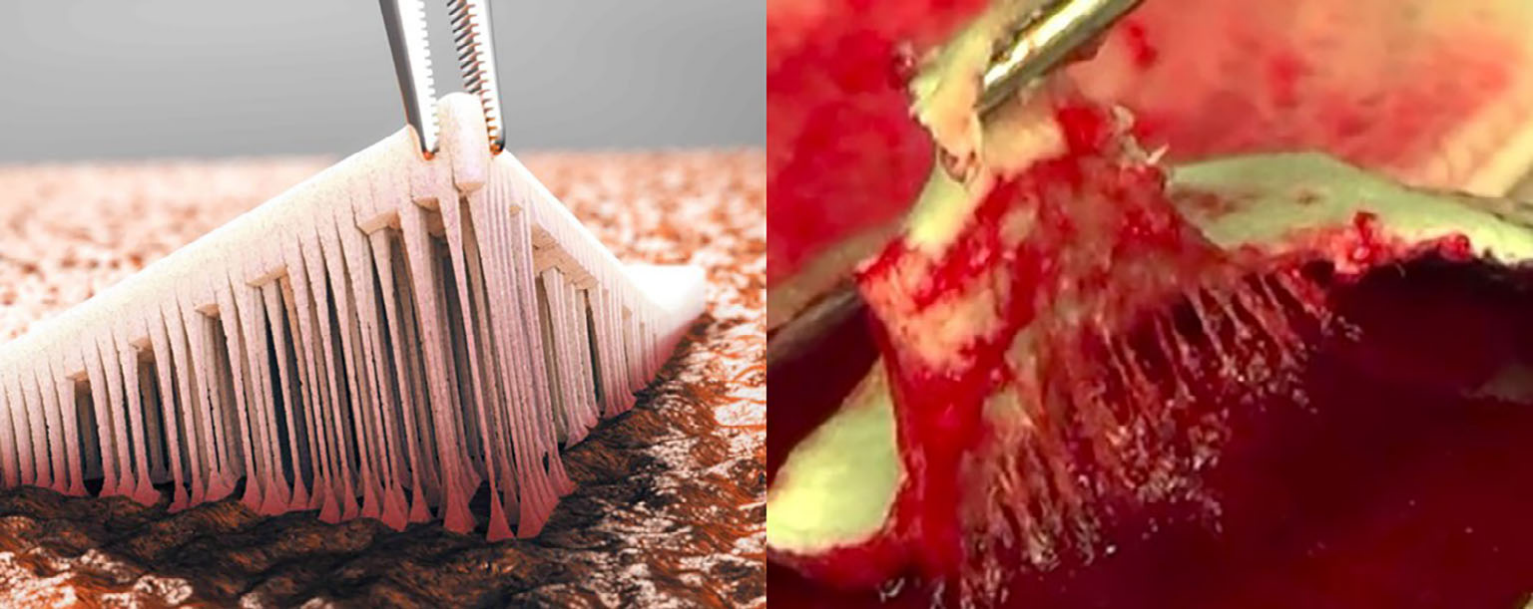
The image on the left represents an animation of the fingerlike projections of the adhesive gelatin (AG) group. The image on the right is a study image demonstrating the adhesive finger‐like projections attaching to the bleeding liver surface.^5^ A video of the procedures can be found here: https://www.youtube.com/watch?v=mpymvGLqL1g. Permission to use this figure has been granted by the copyright holders, Selentus Science Ltd. It was originally published in Dong et al.^5^

Traditional gelatin sponges are inexpensive to manufacture, contain no blood‐derived products and are rapidly absorbed, minimizing the risk of foreign body reactions.^9^ However, they are poorly adhesive to the bleeding tissue, as demonstrated in an in vivo leporine model.^9^ TenaTac (Selentus Science, UK) is a traditional porcine‐gelatin sponge modified to increase surface adhesion. This sponge has modifications on one surface, consisting of over 1000 partial thickness columns, increasing the surface area of the sponge by a factor of 10. These columns are designed to conform to and interact with the variations in tissue surface topography to increase sponge‐tissue contact and adhesion, and to resist shear forces that may dislodge the sponge in the perioperative period. An in vivo leporine‐based study performed by the manufacturers of TenaTac revealed a significant, six‐fold increased tissue adhesion relative to plain gelatin sponge.^9^ Figure 1 demonstrates how the “fingers” of gelatin are designed to interact with variations in surface topography of the bleeding surface and increase tissue adhesion. The adhesive qualities of this product have been proven in an in vivo rabbit model with the improved adhesion linked with the surface modifications of the product, albeit in this laboratory setting, no difference in hemorrhage control was noted between the modified and unmodified gelatin sponges.^9^

This study was designed to compare the modified adhesive gelatin sponge (TenaTac, Selentus Science, Grantham, UK) to the bovine‐collagen hemostatic sponge (Lyostypt, B. Braun, Melsungen, Germany) currently in general use in our hospital. Our aim was to determine whether the modified adhesive gelatin sponge is an effective hemostatic agent in canine liver surgery, and to compare its effectiveness to the bovine‐collagen sponge. The authors hypothesized that the modified gelatin sponge would have greater adhesion and be at less risk of dislodgement than the bovine‐collagen hemostatic sponge, but with no overall difference in bleeding scores between the two sponges.

## 2 MATERIALS AND METHODS

### 2.1 Subjects

The Institutional Committee for Animal Research and Ethics (Nottingham, UK) approved this study (no. 3330 210215) and informed, written client consent was obtained for each dog enrolled. Inclusion criteria were dogs undergoing open liver surgery or liver biopsy for naturally occurring disease, warranting surgical intervention. For case inclusion, the primary surgeon had to identify sufficient parenchymal ooze intraoperatively to warrant placement of hemostatic sponge as defined by hemostatic scoring. Exclusion criteria were as follows: hemodynamic instability preventing safe instigation or completion of the study period; suspected or confirmed coagulopathy; uremia (defined as azotemia with a creatinine level of at least twice the reference interval in a normally hydrated patient and a urine specific gravity of <1.012); administration of anticoagulant medications with the exception of non‐steroidal anti‐inflammatory medications; known or suspected hypersensitivity to collagen or gelatin; presence of a severe comorbidity (American Society of Anesthesiologists physical status grading score >/= 4), administration of immunosuppressive therapy or chemotherapy within the 4 weeks preceding surgery and/or a recorded intraoperative core body temperature of <33°C.

### 2.2 Treatment

Dogs were assigned to either the plain collagen (PC) or the adhesive gelatin (AG) group. The PC group received a sponge manufactured from bovine collagen (Lyostypt, B. Braun), whilst the AG group received a sponge manufactured from porcine gelatin and containing column shaped, surface modifications (TenaTac, Selentus Science). The largest available AG was 8 × 5 cm and the largest PC was 10 × 12 cm. Dogs were assigned utilizing an online stratified block randomization via a text (SMS) messaging service (https://www.sealedenvelope.com/simple-randomiser/v1/lists [Accessed 14 Dec 2020] Seed: 35670988343243, block sizes: 2,4) which was undertaken by a registered veterinary nurse at the time of hemostatic sponge selection. Anesthesia protocols were determined by an anesthetist on an individual basis. Each dog was aseptically prepared for surgery in a standard fashion and 20 mg/kg cefuroxime administered IV approximately 30 min prior to surgery, and then every 90 min until abdominal closure. Routine ventral midline celiotomy was performed in all dogs, and the primary surgical procedure undertaken prior to hemostatic sponge testing. Surgery and hemostatic assessment was performed by five Diplomates of the European College of Veterinary Surgeons. Prior to performing clinical cases, all surgeons were introduced to the methodologies and scoring systems during a short training session and group discussion. Furthermore, all surgeons were highly familiar with the PC sponges, and had used the AG sponge on at least three clinical cases prior to study commencement, in order to become familiar with the product.

Hemostatic sponge testing was performed on a peripheral liver biopsy site or the cut parenchymal surface following liver lobectomy. For liver biopsy, a 15 mm length of liver lobe edge was isolated and two curved mosquito hemostats placed in an overlapping crushing fashion to isolate the segment of liver. The isolated segment was sharply excised with a no. 11 scalpel blade and the mosquito hemostats immediately released. The primary surgeon assessed the cut surface to ensure that a degree of hemorrhage consistent with parenchymal ooze was present. Where liver lobectomy was performed the liver was skeletonized, and a two‐row thoracoabdominal stapler (staple open height 3.5 mm, closed height 1.5 mm) was fired across the larger vessels and bile ducts. The assigned hemostatic sponge for the patient was selected and its size was ensured to achieve 10 mm of coverage beyond the bleeding surface and held in situ with a moist 5 × 5 cm surgical swab for 3 min. Surgeons took care to apply the modified side of the sponge in the AG group against the bleeding parenchymal surface and where necessary the sponge was compressed using gentle digital pressure and/or lightly moistened with sterile saline prior to use to allow the sponge to become more pliable, and accurately conform to the liver edge. This was not required for the sponge in the PC group which was already highly pliable.

### 2.3 Hemostatic scoring

Bleeding scores were obtained from all cases but a group consisting of liver biopsies only is reported independent of the lobectomy cases, as the size of bleeding surface and procedures to obtain the bleeding surface was not homogenous with the liver biopsy cases. For hemostatic testing, following removal of the damp swab, a bleeding score was recorded at time points 3, 4, 5 and 6 min according to a 0–5 ordinal visual bleeding scale where score 0 represented no bleeding, scores 1–5 represented ooze through or peripheral to the hemostatic agent where 1 was minimal, 2 was very mild, 3 was mild, 4 was moderate and 5 was severe (Figure S1). If there was “strike through bleeding” from the hemostatic sponge prior to abdominal closure, a second sponge was applied and held in situ for 1 min and this event was recorded. At the time of hemostatic scoring, blood pressure was recorded from direct arterial pressure monitoring via peripheral arterial cannulation.

### 2.4 Adhesion scoring

Once all bleeding scores were obtained, a 2 × 2 mm full thickness segment at the corner of the hemostatic sponge was grasped with DeBakey thumb forceps and slowly lifted from the liver surface to apply tension to the outer 10 mm of the sponge directly perpendicular to the long axis of the sponge, and the following adhesion scores were assessed: 0–no adhesion; 1–some adhesion, but the sponge quickly lifts away; 2–stronger adhesion but some sponge‐tissue separation occurs and 3–strongly adherent such that the sponge tears when lifted or the organ lifts.

### 2.5 Handling scoring

Surgeons were instructed to grade the handling characteristics of the sponge as:

1. Poor (sponge was stiff and poorly conformed to the tissue, sponge was challenging to apply, sponge adhered to gloves during handling resulting in sponge tearing, sponge was challenging to trim to desired dimensions if required).
2. Satisfactory (sponge was relatively pliable and conformed adequately to tissue, application was straightforward, minor adhesion of sponge to gloves during handling that did not tear the sponge, sponge was able to be trimmed to the required dimensions).
3. Good (sponge pliable and highly conforming to tissue, no adhesion of sponge to gloves during handling, easy to trim to required dimensions).

Following bleeding, adherence and handling scores, the abdomen was lavaged once by filling the abdominal cavity with sterile saline stored in a warming cabinet set to 38°C. Care was taken to ensure that the saline was poured into the mid‐to‐caudal abdomen and not directly onto the liver or sponge. Prior to closure, any further surgical procedures such as active suction drain or gastrostomy tube placement was performed. The sponge was closely monitored for dislodgement before and during linea alba closure, and if noted, sponge dislodgement from the site of bleeding was recorded.

### 2.6 Postoperative management and follow‐up

Postoperative recovery occurred in an intensive care unit staffed by nurses under the direct supervision of a Diplomate of the American College of Veterinary Emergency and Critical Care. As a minimum, the following parameters were measured by the nursing team every 30 min for the first 3 h, hourly for the next 3 h, and then at 9‐ and 12‐h post‐procedure: pulse rate, pulse character, mucous membrane color, capillary refill time, respiratory rate, heart rate and rectal temperature, in order to monitor for both postoperative hemorrhage and allergic reaction to the hemostatic sponge, respectively. If significant alteration in assessed parameters was identified, then this was reported to the duty criticalist. If hemorrhage was suspected, then abdominal point‐of‐care ultrasound and packed cell volume assessment was performed in addition to further testing at the discretion of the duty criticalist and the primary surgeon. Dogs were subsequently monitored based on the primary surgical procedure and discharged from the hospital once it was deemed appropriate by the attending surgeon. Comprehensive written discharge instructions were provided instructing each owner to contact our institution in the event of any adverse events.

Recheck evaluation of the dogs by the primary surgeon was recommended 10–14 days postoperatively. Re‐examination consisted of an updated owner history, general physical examination and examination of the surgical incision. Complications were recorded on practice management software, reported to the principal investigator (TA), and addressed as deemed appropriate.

### 2.7 Statistical analysis

Descriptive statistics focused on the median and range for continuous and ordinal data and percentages for categorical data. Hemostatic parameters are reported as mean. These are summarized for the entire sample, and separately for the two treatments. Responses (adhesion, dislodgement, bleeding score) were compared between the two treatment groups using Fisher's exact tests for binary data, and Mann Whitney U tests, adjusted for ties, for ordinal data. The ordinal bleeding score for biopsy cases was recorded at 3, 4, 5 and 6 min. Bleeding score was analyzed in an ordinal logistic mixed model with time and group as fixed effects and case number as a random effect. At each time point cases were assigned as “bleeding” or “not bleeding” and evaluated with a chi‐squared test. The gradient of each linear trendline was calculated and used to calculate the timepoint at which 50% of cases had stopped bleeding. The model was fitted in the cumulative link mixed model (clmm) routine, and the significance of the group effect was assessed using a likelihood ratio test in the analysis of variance (ANOVA) clmm routine, both in R 4.2.2.

Post hoc power analysis was performed to assess study size and statistical power. Analysis was undertaken by a professional statistician (TS) in Minitab 19 and R 4.2.2. Statistical significance was taken as *p <* .05.

## 3 RESULTS

### 3.1 Subjects

A total of 45 dogs met the inclusion criteria and were randomized to their study group, of which 23 were assigned to the PC group and 22 were in the AG group. No dogs were excluded prior to surgery. One dog randomized to the AG group was diagnosed with metastatic thyroid neoplasia and was subsequently excluded.

### 3.2 Surgery

Surgeries performed in addition to liver biopsy in these cases were cholecystectomy (PC = 9; AG = 8), liver lobectomy (PC = 3; AG = 2), liver lobectomy and cholecystectomy (PC = 0; AG = 1), splenectomy (PC = 3; AG = 2), single extra‐hepatic portosystemic shunt attenuation (PC = 1; AG = 5), gastrointestinal biopsies (PC = 1; AG = 0), partial pancreatectomy (PC = 1; AG = 1) resection of an omental mass (PC = 2; AG = 0), and resection of a renal mass (PC = 0; AG = 1). The pathological diagnoses of these cases are recorded in Table S1. For the cases undergoing only liver lobectomy (PC = 3; AG = 2), no concurrent liver biopsy was performed and the site of testing was the lobectomy site.

No case was hypotensive (MAP < 50 mmHg) at the time of biopsy, with group PC having a median mean arterial pressure of 78 mmHg (range: 60–125), and group AG a median mean arterial pressure of 84 mmHg (range: 60–120) (*p* = .872).

### 3.3 Adhesion and handling scores

**FIGURE 2:**
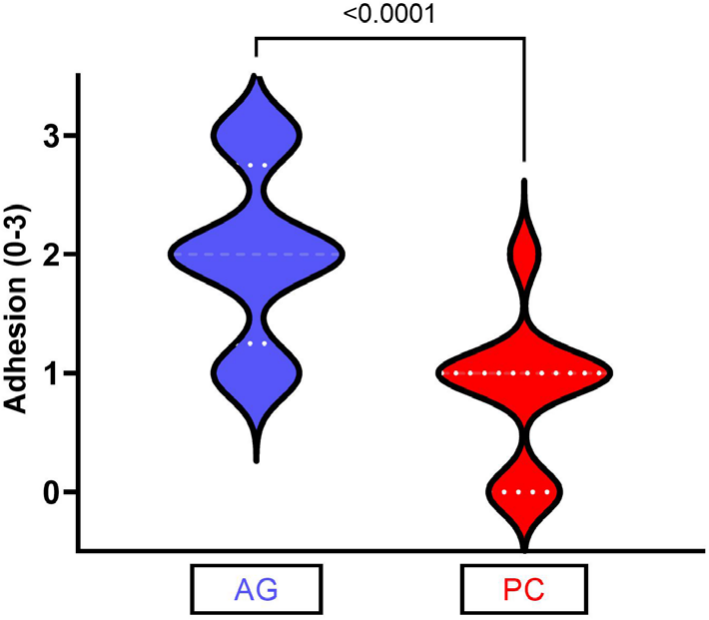
The level of adherence to the liver was graded out of three by the primary surgeon. The adhesive gelatin (AG) group sponges (*n* = 23) were significantly more adhesive than the plain collagen (PC) group (*n* = 22) (*p* < .001).

Sponge adhesion and handling was assessed in all cases (*n* = 45). Median sponge adhesion was significantly greater in the AG group (2 [1–3]) than the PC group (1 [0–2]) (*p* < .001*) (Figure 2). None of the AG group scored zero in the adhesion assessment (0/22), compared to 6/23 in the PC group (*p* = .022). Conversely, when the highest half of the adhesion scores were measured (2 or 3), 17/22 AG cases fell into this category, as opposed to 3/23 for the PC group (*p* < .0001). No difference was reported in the handling scores of the two sponges (PC = 2 (1–3); AG = 2 (1–3); *p* = .496).

Post hoc powering for a Mann Whitney U test of the adhesion score of an effect size of 1.0, a pooled standard deviation of 0.684, and a significance level of 0.05 was based on powering for a two‐sample *t*‐test with a 15% uplift in sample size. This generated a post hoc powering for the liver biopsy (20 AG, 20 PC) of 0.98, and for the full dataset (22 AG, 23 PC) of 0.99.

### 3.4 Hemostasis

**FIGURE 3:**
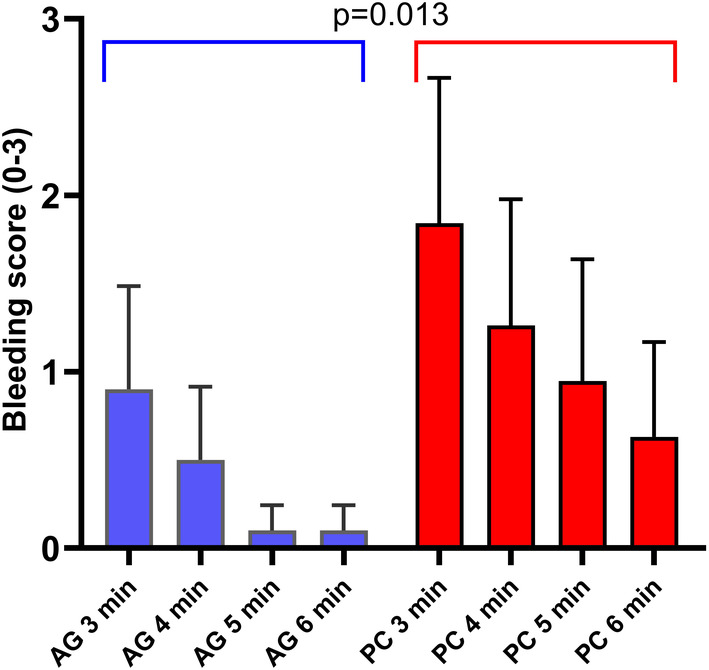
The bleeding severity scores were assessed by the primary surgeon against a visual scoring chart. The mean values (+SD) are shown with the adhesive gelatin (AG) group exhibiting significantly lower levels of bleeding when evaluated across the four time points (*p* = .013 in an ordinal logistic mixed model). Use of mean rather than median values is used in this figure to highlight differences between the groups; however, it should be noted that the data is not normally distributed.

The ordinal logistic mixed model performed on the cases from which bleeding scores were obtained from the biopsy site (*n* = 40), revealed significantly lower bleeding scores in the AG group (chi‐square [χ^2^] = 6.11, *p* = .013*) (Figure 3). The severity of the mean bleeding scores were generally low throughout for both groups beyond 4 min, yet significantly higher for the PC group at 5 min (*p* = .027) relative to the AG cohort (Table 1). The biopsy plus lobectomy cases (*n* = 45) are also shown in Table 1, with the severity of the bleeding scores significantly higher for the PC group at 5 (*p* = .005) and 6 min (*p* = .034) relative to the AG cohort. Post hoc powering of the bleeding scores at each time point, using the observed differences and standard deviations, following the approach used for adhesion powering above, generated power values ranging from 0.45 to 0.78 (mean, 0.68).

**TABLE 1:** Mean bleeding scores, range and interquartile range at each time point for the biopsy cases alone and then all cases.

Biopsy only	3 min	4 min	5 min	6 min
AG mean (*n* = 20)	0.9	0.5	0.1	0.1
AG range	0–3	0–3	0–1	0–1
AG IQR	0–2	0–1	0–0	0–0
PC mean (*n* = 20)	1.8	1.2	0.9	0.6
PC range	0–4	0–4	0–4	0–3
PC IQR	0–3.5	0–2.5	0–2	0–1
AG vs. PC (*p* =)	.0596	.0895	.0272	.0735

All cases	3 min	4 min	5 min	6 min
AG mean (*n* = 22)	1.0	0.5	0.1	01
AG range	0–3	0–3	0–1	0–1
AG IQR	0–2	0–1	0–0	0–0
PC mean (*n* = 23)	1.9	1.4	1.0	0.7
PC range	0–4	0–4	0–4	0–3
PC IQR	0–3.5	0–3	0–2	0–1
AG vs. PC (*p* =)	.0550	.0516	.0048	.0335

Abbreviations: AG, adhesive gelatin; IQR, interquartile range; PC, plain collagen. Higher bleeding scores indicate poorer hemostasis. 0 represented no bleeding, scores 1–5 represented ooze through or peripheral to the hemostatic agent where 1 was minimal, 2 was very mild, 3 was mild, 4 was moderate and 5 was severe. Use of mean rather than median values is used to highlight differences between the groups; however, it should be noted that the data is not normally distributed.

**FIGURE 4:**
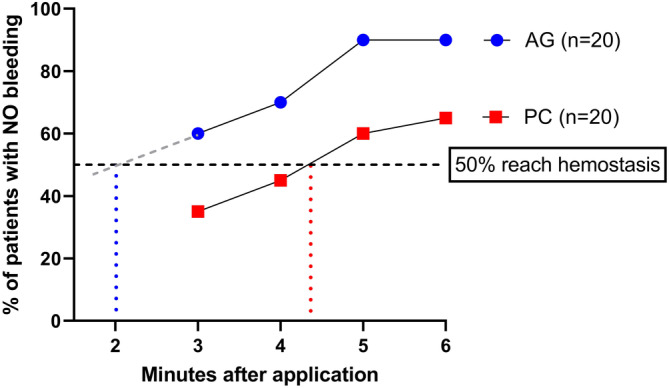
The proportion of dogs in each liver biopsy only group (*n* = 40) where the bleeding had stopped completely at the four time points assessed. The vertical blue and red dotted lines indicate the time taken for 50% of the cases to reach hemostasis.

As well as the severity of the bleeding, the number of dogs obtaining full hemostasis was recorded (*n* = 45). At 3 min, 15/23 patients in PC were still bleeding, compared to 10/22 in AG (*p* = .465). At 5 min the difference between the groups was more pronounced with 10/23 still bleeding in the PC arm, compared to 2/22 in AG arm (*p* = .009). Figure 4 displays the full data. The gradient of each linear trendline was calculated and used to calculate the timepoint at which 50% of cases had stopped bleeding. The time for the AG group was 2 min 00 s compared to 4 min 49 s for the PC group.

A second sponge was applied in seven of 45 cases (PC = 6; AG = 1; *p* = .182). The sponge dislodged in six cases (PC = 6; AG = 0; *p* = .022*), all of which occurred during abdominal lavage and all of which led to mild secondary hemorrhage from the biopsy site and required placement of a second sponge. In one AG case, the primary surgeon elected to place a second sponge at 6 min post biopsy for the management of strike through bleeding, albeit this was visually graded only as a grade 1 (minimal). Post hoc powering for a Fisher's exact test of the dislodgement frequencies, based on the observed frequencies and a significance level of 0.05 generated a power value of 0.58.

### 3.5 Adverse events

No patient had a variation in monitored parameters which was attributed to postoperative hemorrhage during the immediate postoperative recovery period. No complication attributed to the use of a hemostatic sponge was noted.

## 4 DISCUSSION

This prospective, randomized study sought to determine the effectiveness and adhesion of a modified porcine‐derived gelatin (AG) hemostatic sponge and compare it to a bovine‐derived collagen (PC) sponge during canine liver surgery. We partially accept our hypothesis since AG had significantly greater adhesion and less dislodgement; however, it was also associated with reduced bleeding score. Adhesion is of benefit in many surgical applications, especially within body cavities where poor adhesion and sponge dislodgement could result in delayed hemorrhage.^9^ In the collagen group, second sponges were used in six cases where sponge dislodgement occurred. A second sponge was only required once in the AG group due to bleeding through the hemostatic sponge, and there were no dislodgements seen in this cohort. The findings of this study are consistent with a recent cohort analysis of the effectiveness of AG across a wide range of human surgical procedures.^11^ That clinical study of a variety of surgical cases reported that AG demonstrated “excellent” or “very good” adhesion in 83% of cases and the hemostasis was rated similarly in 92% of cases, by human consultant surgeons.^11^

Collagen sponges are reported to be a superior hemostatic agent, attributed to greater platelet activation and clot formation than gelatin sponges in vitro,^3^ although when in vivo platelet activation was tested in a human model, there were no differences noted between collagen and gelatin.^12^ Similar findings were noted in an experimental study utilizing a rat liver bleeding model, in which no difference in the hemostatic properties of gelatin or collagen sponges were identified.^13^ In our study we found significantly decreased bleeding scores attributed to the gelatin sponge (AG) compared to the collagen sponge (PC) both in terms of the severity scores, as well as the number of patients in whom complete hemostasis was obtained. The reason for this outcome probably lies with the increased adhesion to the bleeding surface and increased sealant/tamponade effect, with the gelatin sponge compared to the collagen sponge. Importantly, overall hemostatic scores, especially at 5–6 min post‐sponge placement, were limited to mild or very mild in both study groups.

In one study, the use of a bovine‐gelatin and human‐thrombin matrix for hemostasis following canine hepatic biopsy led to significantly reduced blood loss, and significantly shorter time to hemostasis than a standard gelatin sponge. The authors of this study attributed this to superior conforming and adherence of the matrix to the bleeding surface, as well as the addition of human thrombin.^14^ Whilst thrombin containing hemostatic agents may be superior, especially in patients with coagulopathies where they do not require a fully intact coagulation cascade to be efficacious, they are expensive, classified as drugs making their handling more complex, and the canine immunogenicity of human thrombin is unknown, potentially increasing the risk of anaphylactic reactions.^15^

A previous retrospective study investigated the use of porcine‐gelatin hemostatic sponges in small animals, with no hypersensitivity or postoperative complications reported within the follow‐up period of a median of 13 months.^16^ This current study supports their findings regarding intraoperative safety, albeit due to the relatively short follow‐up time, and the primary focus on effectiveness of the sponges at hemostasis, reporting on the safety of either the gelatin or collagen sponge is beyond the scope of this study.

### 4.1 Limitations

Although this study represents the first randomized study of hemostatic agents within the veterinary literature, it inevitably had some limitations. It did not have a negative control group and it is therefore possible that hemostasis would have been achieved without the use of a hemostatic agent by allowing the surface to bleed and clot, or the application of digital pressure only. We did not test a plain gelatin sponge comparative group, as previous laboratory experiments have demonstrated this modified gelatin sponge exhibits greater adhesion than a plain gelatin sponge in a leporine liver model.^8^ Whilst assessing hemorrhage for a modified versus unmodified gelatin sponge would have been interesting, the primary clinical problem of interest was sponge dislodgement, and we sought to evaluate the product which was most likely to reduce this concern. Furthermore, a previous study comparing a plain gelatin sponge to a plain collagen sponge in dogs undergoing craniotomy found the gelatin sponge to be less effective at hemostasis, attributing this to poor adhesion to the underlying bleeding tissue, albeit this study is not directly comparable to the use of the sponge in hepatic surgery, as in our study.^17^ Our methodology applied the hemostatic sponge to the surface of the liver, covering the bleeding surface and relying on adhesion between the liver surface and sponge to prevent sponge dislodgement. Use of the punch biopsy technique for liver biopsy, where a small piece of hemostatic sponge can be rolled and placed into the punch hole, may remove the benefit of adhesive hemostatic agents during liver biopsy, as hemostatic sponge placement is more secure. This technique was not performed in our study, and further studies would be warranted to assess the potential benefit of an adhesive sponge for this application.

A further limitation of this study was the heterogenous population of dogs and indication for surgery. The use and strict adherence to stratified block randomization was designed to limit confounding variables to some degree. We did not ultrasound the site of testing in the postoperative period so low volume hemorrhage insufficient to result in systemic signs may have been overlooked. Our study included five extrahepatic portosystemic shunt (EHPSS) cases in group AG compared to one in group PC. Patients with EHPSS have previously been reported to have coagulation abnormalities including lower platelet counts, lower activity of factors II, V, VII, and X, increased factor VIII and prolonged activated prolonged partial thromboplastin time prior to shunt attenuation, and decreased platelet counts and activity of factors I, II, V, VII, IX, X, and XI and a prolonged prothrombin time following attenuation.^18^, ^19^ Despite these biochemical changes, in the study by Niles et al. ^19^ there was no increased bleeding tendency identified. Whilst it is a limitation that there are not equal number of EHPSS in both groups, shunts likely represent on average prolonged coagulation with no clinical bleeding tendency identified, and these cases were more prevalent in the adhesive gelatin group, rather than the plain collagen. All patients received a single lavage to fill the abdominal cavity prior to closure; however, we did not standardize lavage volume, or time from biopsy to completion of abdominal closure. The effect of variations in these parameters outside of the direct study measurements should be limited by strict adherence to randomization. We also included patients which had undergone stapled liver lobectomy and, like cases reported previously, these cases had undergone skeletonization and application of a thoracoabdominal stapler but had ongoing diffuse hemorrhage.^20^, ^21^ Whilst we have been careful to calculate a bleeding score excluding these more heterogenous cases, we believe they represent a relevant and real‐world application of hemostatic sponge use.

We did not use a validated bleeding scale for this study. Whilst a study protocol for human hepatic intraoperative bleeding scale validation has been published and began recruiting cases in September 2023, it is yet to be published.^22^ It may be possible to adapt this scale for dogs ensuring that a validated scale exists for future studies involving canine hepatic hemorrhage. A further limitation of this study was that it was not blinded, and this allows the potential for individual bias as surgeons may prefer one hemostatic agent over another. There were five individual surgeons performing surgeries and collecting data. This may allow interobserver variability reducing the precision of scoring; however, the use of multiple surgeons may also mitigate any individual bias. In addition, whilst knowledge of coagulopathy was an exclusion criterion, we did not require advanced coagulation testing prior to inclusion in the study. Thromboelastographic analysis would be considered the gold standard test to exclude coagulopathy but would be unlikely to be used in the general referral setting for this population of patients and therefore was not required for study inclusion.^23^ This raises the possibility that the differences observed in bleeding score may be due to differences in coagulation; however, the use of block stratified randomization should ensure that a potential baseline confounding variable is distributed between the two groups. We acknowledge that whilst we performed power calculations, the relatively small sample size may have led to statistical errors.

## 5 CONCLUSION

In these clinical cases of mild or very mild intraoperative hepatic parenchymal oozing, a novel adhesive gelatin sponge created less dislodgement and improved hemostasis compared to plain collagen.

## AUTHOR CONTRIBUTIONS

Anderson TS, BVSc, PGCert, DipECVS, MRCVS: Contributed to the design of the study, oversaw data collection, provided interpreted data and provided scientific, in‐line editing of the manuscript. Hattersley RD, BVetMed (Hons), CertSAS, DipECVS, MRCVS: Contributed to case acquisition and the surgical management of cases, and provided scientific, in‐line editing of the manuscript. Demetriou JL, BVetMed, CertSAS, DipECVS, FRCVS: Contributed to the design of the study, was responsible for the surgical management of cases, and provided scientific, in‐line editing of the manuscript. All authors provided a critical review of the manuscript and endorse the final version. All authors are aware of their respective contributions and have confidence in the integrity of all contributions.

## CONFLICT OF INTEREST STATEMENT

None of the authors had any conflict of interest to declare. The adhesive gelatin sponges (TenaTac) were donated by Selentus Science Ltd.

## Supporting information


**Figure S1.** Visual scoring chart provided to surgeons for hemostatic scoring.

**Table S1.** Histological diagnoses.

## Data Availability

The data that support the findings of this study are available from the corresponding author upon reasonable request.
