# Supplementary figures and images for: A randomized comparison of an adhesive gelatin sponge and a plain collagen sponge for hemostatic control during canine liver surgery
Source: Vet Surg. 2024 Oct 8;54(2):345–53. doi: 10.1111/vsu.14160 (PMC11830848; doi:10.1111/vsu.14160)

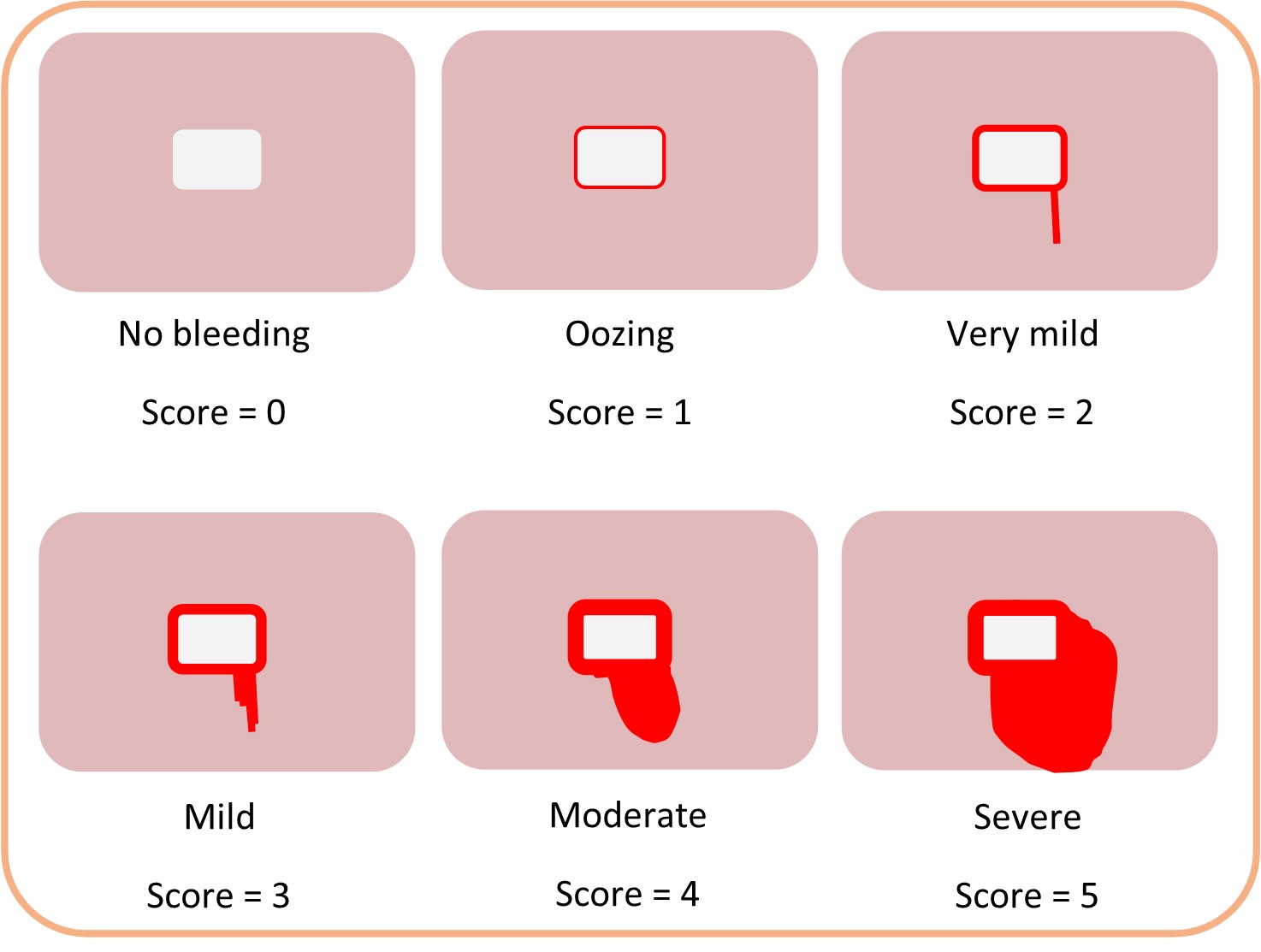

Supplement: Supplementary file 1 — Figure S1. Visual scoring chart provided to surgeons for hemostatic scoring. [file VSU-54-345-s001.jpg]
