# Supplementary material for: A randomized comparison of an adhesive gelatin sponge and a plain collagen sponge for hemostatic control during canine liver surgery
Source: Vet Surg. 2024 Oct 8;54(2):345–53. doi: 10.1111/vsu.14160 (PMC11830848; doi:10.1111/vsu.14160)
Supplement: Supplementary file 2 — Table S1. Histological diagnoses. [file VSU-54-345-s002.docx]

Supplementary table 1 – histological diagnoses

| **Plain collagen cases** | **Adhesive gelatin cases** |
| --- | --- |
| **Liver biopsy cases** | **Liver biopsy cases** |
| Hepatocellular hydropic degeneration and vacuolation, chronic, diffuse; with mild ductular reaction. | Moderate multifocal predominantly midzonal, hepatocellular swelling and clearing. There are rare lymphocytes and neutrophils within portal areas. |
| Focal nodular hyperplasia | Moderate diffuse mixed portal hepatitis with moderate biliary hyperplasia, moderate fibrosis, mild cholangitis and moderate vacuolar hepatopathy |
| Hepatocellular lipidosis and mild neutrophilic portal infiltrates | Portal hepatitis, neutrophilic, chronic, diffuse, mild to moderate. |
| Liver tissue containing a neoplastic nodule of likely endocrine/neuroendocrine (most likely metastasis of insulinoma) | Hepatitis, portal and periacinar, neutrophilic, eosinophilic, lymphocytic and plasmacytic, minimal to mild, oedema, mild periductal fibrosis and vacuolar hepatopathy, periportal, mild to moderate. |
| Mild to moderate lymphocytic and neutrophilic portal hepatitis with mild to moderate biliary hyperplasia | Diffuse moderate arteriolar portal hyperplasia and mild biliary hyperplasia |
| Hepatocellular hydropic degeneration and pigmentation and chronic, diffuse, moderate mild portal hepatitis associated with lymphoplasmacytic, neutrophilic, multifocal, mild and pigmented pyogranulomas. | Consistent with focal hyperplasia and vacuolated hepatopathy  Lobular hepatic hypoplasia with moderate arteriolar hyperplasia and apparent loss of venous profiles |
| Moderate portal arteriolar hyperplasia and mild biliary hyperplasia | Moderate vacuolar hepatopathy and mild bile duct dilation |
| Hepatocellular hydropic degeneration and mild pigmentation, chronic, centrilobular to diffuse | Suspect focal nodular hyperplasia |
| Vacuolar hepatopathy (hydropic degeneration or glycogen accumulation) and mild multifocal neutrophilic and lymphoplasmacytic portal hepatitis | Portal ductular reaction, chronic, mild; Portal mixed inflammatory infiltrated, chronic, mild; Portal fibrosis, chronic, minimal; increased numbers of foamy macrophages and circulating neutrophils, subacute/chronic, mild; hepatocellular hydropic degeneration, multifocal, subacute/chronic, mild; and hypertrophy and hyperplasia of hepatic stellate cells, diffuse, subacute/chronic mild. |
| Portal ductular reaction and intrabiliary mucinous content, chronic, diffuse, mild to moderate; and portal hepatitis, neutrophilic, | Cholangiohepatitis, neutrophilic, lymphocytic, plasmacytic, chronic, diffuse, mild to moderate, with biliary hyperplasia, peribiliary fibrosis, hepatocellular degeneration, diffuse, mild to moderate; liver. |
| Cholangiohepatitis, neutrophilic, lymphocytic, plasmacytic, chronic, multifocal, mild, with biliary hyperplasia, multifocal, mild; liver | Portal vein hypoplasia and arteriolar duplication with vacuolar hepatopathy, periacinar to midzonal, mild to moderate. |
| Suspect focal nodular hyperplasia with hepatocellular cell swelling. | Cholangitis, neutrophilic, lymphocytic and plasmacytic, mild to moderate, chronic with mild periductal fibrosis and mild bile ducts hyperplasia. |
| Hepatitis, portal, neutrophilic, lymphoplasmacytic, chronic, multifocal, minimal | Hepatocellular atrophy, chronic, diffuse; with arteriolar reduplication. |
| Hepatitis, portal, neutrophilic, chronic, multifocal, minimal | well-differentiated hepatocellular proliferation has features primarily consistent with a focus of hepatocellular nodular hyperplasia |
| Hepatitis, neutrophilic, mild, multifocal, acute with cloudy swelling, mild to moderate, diffuse. | Portal vein hypoplasia, with arteriolar proliferation, endothelial cell hyperplasia and lobular atrophy, diffuse, moderate; liver. |
| vacuolar hepatopathy, subacute/chronic, diffuse, moderate | Mild multifocal periportal hepatocellular vacuolar hepatopathy |
| Forming a focal non encapsulated lesion, disrupting hepatocytes plates, the sinusoids are dilated and hepatocytes plates are lined by prominent plump endothelial-like cells. | Focal suppurative hepatitis |
| Hepatitis, portal, lymphocytic, plasmacytic, neutrophilic, mild to moderate, chronic with portal fibrosis, moderate, chronic multifocally bridging porto-portal, with periductal fibrosis, mild, and bile ducts hyperplasia, moderate, with extramedullary haematopoiesis, mild, multifocal and mild fibrinoneutrophilic serositis | Diffuse, severe, vacuolar hepatopathy with foci of acute haemorrhage/fibrin accumulation/thrombosis |
| Consistent with focal nodular hyperplasia; hepatocellular vacuolation/hydropic degeneration, subacute/chronic, diffuse, moderate; hepatitis, portal, neutrophilic, lymphoplasmcytic, subacture/chronic, multifocal, mild. | Nodular hyperplasia with mild to moderate multifocal vacuolar hepatopathy and mild to moderate multifocal extramedullary haematopoiesis |
| Cholangiohepatitis, neutrophilic, chronic, moderate, with mild cholestasis. | Hepatitis, portal, neutrophilic, lymphoplasmacytic, chronic, multifocal |
| **Liver lobectomy cases** | **Liver lobectomy cases** |
| Marked vascular congestion with centrilobular to midzonal predominant, marked hepatocellular atrophy, degeneration and loss (consistent with liver lobe torsion, subacute to chronic) | Consistent with carcinoma, mixed hepatocellular and cholangiocellular |
| Increased biliary ductal elements and arterioles, chronic, diffuse, mild to moderate; with moderate biliary duct dilation, mild fibrosis and minimal sacculation | Well-differentiated hepatocellular neoplasm, favour adenoma. Neoplastic cells extend to the inked margin |
| Hepatocellular carcinoma, well differentiated. 15mm margin of normal parenchyma |  |

Supplementary Figure 1: Visual scoring chart provided to surgeons for hemostatic scoring
